# Mechanism of Porcine Liver Xanthine Oxidoreductase Mediated *N*-Oxide Reduction of Cyadox as Revealed by Docking and Mutagenesis Studies

**DOI:** 10.1371/journal.pone.0073912

**Published:** 2013-09-09

**Authors:** Chigang Chen, Guyue Cheng, Haihong Hao, Menghong Dai, Xu Wang, Lingli Huang, Zhenli Liu, Zonghui Yuan

**Affiliations:** National Reference Laboratory of Veterinary Drug Residues (HZAU) and MOA Key Laboratory for the Detection of Veterinary Drug Residues in Foods, Huazhong Agricultural University, Wuhan, Hubei, China; University of Edinburgh, United Kingdom

## Abstract

Xanthine oxidoreductase (XOR) is a cytoplasmic molybdenum-containing oxidoreductase, catalyzing both endogenous purines and exogenous compounds. It is suggested that XOR in porcine hepatocytes catalyzes the *N*-oxide reduction of quinoxaline 1,4-di-*N*-oxides (QdNOs). To elucidate the molecular mechanism underlying this metabolism, the cDNA of porcine XOR was cloned and heterologously expressed in *Spodoptera frugiperda* insect cells. The bovine XOR, showing sequence identity of 91% to porcine XOR, was employed as template for homology modeling. By docking cyadox, a representative compound of QdNOs, into porcine XOR model, eight amino acid residues, Gly47, Asn352, Ser360, Arg427, Asp430, Asp431, Ser1227 and Lys1230, were located at distances of less than 4Å to cyadox. Site-directed mutagenesis was performed to analyze their catalytic functions. Compared with wild type porcine XOR, G47A, S360P, D431A, S1227A, and K1230A displayed altered kinetic parameters in cyadox reduction, similarly to that in xanthine oxidation, indicating these mutations influenced electron-donating process of xanthine before subsequent electron transfer to cyadox to fulfill the *N*-oxide reduction. Differently, R427E and D430H, both located in the 424–434 loop, exhibited a much lower K_m_ and a decreased V_max_ respectively in cyadox reduction. Arg427 may be related to the substrate binding of porcine XOR to cyadox, and Asp430 is suggested to be involved in the transfer of electron to cyadox. This study initially reveals the possible catalytic mechanism of porcine XOR in cyadox metabolism, providing with novel insights into the structure-function relationship of XOR in the reduction of exogenous di-*N*-oxides.

## Background

Xanthine oxidoreductase (XOR) is a molybdo-flavoenzyme, existing as two forms, xanthine dehydrogenase (XDH; EC 1.17.1. 4) and xanthine oxidase (XO; EC 1.17.3.2) [1]. XOR plays an important role in the catabolism of purines in mammalians. This enzyme catalyzes the oxidation of hypoxanthine to xanthine and further catalyzes the oxidation of xanthine to uric acid. XOR is synthesized as its prevalent XDH form, and can be converted to XO form, either reversibly by disulfide formation or irreversibly by proteolytic cleavage [2]. XOR is involved in many physiological and pathological processes. For example, XOR functions as a source of reactive oxygen species (ROS), which are pathogenic agents in ischemia-reperfusion injury [3]. XOR also plays critical roles in the metabolism of exogenous compounds as one of the most important drug-metabolizing enzymes in addition to cytochrome P450 enzymes. In the presence of an adequate electron donor, XOR can mediate the reduction of various compounds, such as nitrobezoic acid, 1-nitropyrene, 2-nitrofluorene, 4-nitroquinoline *N*-oxide, nitrofurazone and nicotinamide *N*-oxide [1].

XOR comprises two identical subunits with molecular weight of 145 kDa, each of which contains two nonidentical iron-sulfur redox clusters (Fe/S I and Fe/S II), one flavin adenine dinucleotide (FAD) cofactor and a single molybdopterin (Mo-pt) center from N to C terminus [4]. Electrons enter at the Mo-pt center, proceed via two iron-sulfur clusters, and ultimately reach the FAD cofactor. Crystal structures of the bovine XOR in complex with the physiological substrates of xanthine [5] and hypoxanthine [6], the chemotherapeutic agents of lumazine [5] and 6-mercaptopurineat [6], the poor substrate of 2-hydroxy-6-methylpurine [7], and the inhibitor of FYX-051 [8] have been determined, demonstrating the bindings and the orientations of the substrates in the active sites of Mo-pt center, in which Glu802 and Arg880 (bovine numbering) are universally conserved. Physiologically, the most pronounced difference between XDH and XO is the terminal electron acceptor to FAD, as XDH strongly prefers nicotinamide adenine dinucleotide (NAD^+^) over molecular oxygen (O_2_), while XO can only use O_2_ [9]. Recently, the XDH crystallized in complex with NAD^+^ has been solved [2]. Although the conformation change of 423–433 loop (bovine numbering) near the flavin binding site is important for the conversion from XDH to XO [10], conformational change in this loop region was not observed in response to NAD^+^ binding [2].

Quinoxaline-1,4-di-*N*-oxides (QdNOs) have shown manifold biological properties, including antibacterial, antiviral, anticancer, antifungal, and insecticidal activities [11]. Cyadox, a QdNO derivative, is a novel antimicrobial and growth-promoting agent used in feed. It was reported that cyadox exhibited less toxicity, greater growth-promoting effect and higher antimicrobial activity, compared with those of other QdNO growth promoters [12]–[15]. Both *in vivo* and *in vitro* studies showed that cyadox was *N*-oxide reductively metabolized in rat, chicken and swine [16]–[19]. Recent study demonstrated that cyadox could be enzymatically reduced to cyadox-4-monoxide and cyadox-1-monoxide, catalyzed by aldehyde oxidase and XOR in the cytosol, and by cytochrome b5 reductase in the microsomes [20]. *In vitro* study demonstrated that QdNOs caused hypoxia-selective DNA cleavage by redox-activation under xanthine/XOR system [21]. Till now, there is no study about the catalytic mechanism of XOR in the reduction of exogenous compounds of di-*N*-oxides. Since cyadox is mainly used in swine, and swine is one of the most important food producing animals as well as a useful model for medical research [22], it is necessary to investigate the catalytic mechanism of porcine XOR in the reductive metabolism of cyadox.

Molecular docking is a method which predicts the preferred orientation of one molecule to a second when bound to each other to form a stable complex [23]. Site-directed mutagenesis of a key amino acid residue may result in property changes of the protein, affecting its function correspondingly. By combined use of these two approaches, several structure-function relationships of XOR have been explained. For example, inhibitors were docked into the active site of the bovine milk XDH to study the structure-activity relationship between the inhibitory features and the different docking energies [24]. By mutating Cys43, Cys51 (ligands to the Fe/S II) and Cys115 (ligand to Fe/S I) of rat XOR, the sequence of the intramolecular electron transfer was identified occurring as follows: Mo-pt→Fe/S I→Fe/S II→FAD [25]. By site-directed mutagenesis, Trp335 and Phe336 were demonstrated to be important residues for converting rat XOR to highly superoxide-productive XO [26].

In this study, cyadox was chosen as the substrate to investigate the catalytic mechanism of porcine XOR in the reduction of an exogenous electron acceptor. Given that the porcine XOR gene is not available in GenBank database, the cDNA of this gene was cloned and heterologously expressed in *Spodoptera frugiperda* (Sf9) insect cells. The homology modeling of porcine XOR was conducted using the reported crystal structure of bovine milk XOR as template. Molecular docking between porcine XOR as the receptor and cyadox as the ligand was performed to discover the possible key amino acid residues responsible for the binding or catalyzing of cyadox by XOR. These residues were then mutated by site-directed mutagenesis technology. The enzymatic activities of the wild type porcine XOR and its mutants for the physiological substrate, xanthine, and the exogenous substrate, cyadox, were analyzed. It was demonstrated that Arg427 and Asp430 respectively determined the substrate binding and electron transfer process during the reduction of cyadox. This study not only gives insights into the metabolic process of cyadox in swine that is associated with the pharmacological and toxicological activities of this compound, but also enlarges the knowledge about the structure-function relationship of XOR in the metabolism of exogenous compounds.

## Materials and Methods

### Ethics Statement

Animal procedure was carried out in strict accordance with the recommendations in the guidelines of the Committee on the Care and Use of Laboratory Animals of China. The protocol was approved by the Ethical Committee of the Faculty of Veterinary Medicine (Huazhong Agricultural University).

### Chemicals

Cyadox (C_12_H_9_N_5_O_3_, 99.8%) and cyadox-1-monoxide (C_12_H_9_N_5_O_2_, 99.5%) were synthesized by Institute of Veterinary Pharmaceutical of Huazhong Agricultural University (Wuhan, China). Xanthine and uric acid were purchased from Sigma (Saint Louis, USA). The restriction enzymes were purchased from Takara (Dalian, China). All other chemicals and reagents commercially available were of the highest analytical grade.

### Animal, cells and plasmids

4 month old male healthy swine (Danish Landrace × Yorkshire × Duroc crossbreed) was purchased from the China Swine Breeding and Test Center (Wuhan, China). The swine was fed with a basal diet [18] containing no antimicrobial agents/compounds and was acclimatized for 1 week prior to starting the experiment. The swine had access to water *ad libitum*. The environmental temperature of the rooms was controlled at approximately 20 °C.

*Esherichia coli* DH 5α, *E. coli* DH10Bac and pMD 18-T vector were purchased from Takara (Dalian, China). *Spodoptera frugiperda* (Sf9) cells and baculovirus transfer vector pFastBac HTb were purchased from BD Gentest (New Jersey, USA).

### DNA manipulation, plasmid construction and mutagenesis

After 12 hours fast, the swine was exsanguinated by jugular artery bleeding. Swine liver was obtained and stored at −70°C until use. Total RNA was isolated from swine liver cells using TRIzol® Reagents (Invitrogen) following the manufacturer's instructions. The concentration and purity of RNA were estimated based on the absorbances at 260 and 280 nm. PCR primers were designed according to the conservative DNA sequences of XORs from rat [27], mouse [28], human [29], bovine milk [30] and cat [31] (Table 1).

**Table 1:** Primers for porcine XOR cDNA amplification and site-directed mutagenesis.

Primer	Sequence (from 5′ to 3′)
XOR1	Fwd: GACCCCCAACCTGTTGACAATG
	Rev: GTACAACTCGCCTGAAACAACATTA
XOR2	Fwd: TCCAGCCATCATCACAATTGAGGAT
	Rev: CTGCACCTCTCACACATTCACGTTC
XOR^a^	Fwd: GCAGTCGAC **ATG**ACAGCAGATGAGT
	Rev: GGGGTACC **TCA**GACCCTCAGAGAC
G47A^b^	Fwd: CTGTGGAGAAGGG***GCC***TGCGGAGCATGCA
	Rev: TGCATGCTCCGCA***GGC***CCCTTCTCCACAG
N352A^b^	Fwd: GTCCATCGGAGGG***GCC***ATCATCACCGCCA
	Rev: TGGCGGTGATGAT***GGC***CCCTCCGATGGAC
S360P^b^	Fwd: CCGCCAGCCCCATC***GCC***GACCTCAACCCTGT
	Rev: ACAGGGTTGAGGTC***GGC***GATGGGGCTGGCGG
R427E^b^	Fwd: CAAGCAGGCCTCC***GAG***AGAGAAGATGACA
	Rev: TGTCATCTTCTCT***CTC***GGAGGCCTGCTTG
D430H^b^	Fwd: CTCCCGGAGAGAA***CAT***GACATAGCGAAGG
	Rev: CCTTCGCTATGTC***ATG***TTCTCTCCGGGAG
D431A^b^	Fwd: CCCGGAGAGAAGAT***GCC***ATAGCGAAGGTGAC
	Rev:GTCACCTTCGCTAT***GGC***ATCTTCTCTCCGGG
S1227A^b^	Fwd: CACCCGCGGCCCC***GCT***ACCTACAAGATCC
	Rev: GGATCTTGTAGGT***AGC***GGGGCCGCGGGTG
K1230A^b^	Fwd: CCCCAGCACCTAC***GCT***ATCCCTGCCTTCG
	Rev: CGAAGGCAGGGAT***AGC***GTAGGTGCTGGGG

a Primers for wild type porcine XOR cDNA amplification. The restriction sites of *Sal* I and *Kpn* I are underlined in the forward and reward primer, respectively. The start and stop codons are in bold.

b Primers for site-directed mutagenesis. The mutated sites are underlined in bold and italic.

**Figure 1:**
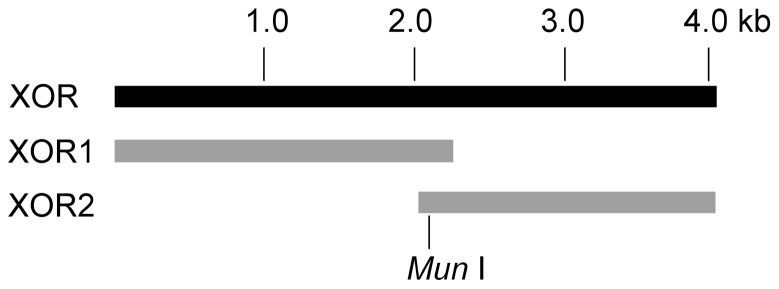
Cloning of porcine XOR cDNA.

To construct the full-length cDNA of porcine XOR, two cDNA fragments, XOR1 (containing nucleotide 1–2211) and XOR2 (containing nucleotide 2082–4005) (Figure 1) were obtained by reverse transcription of total RNA extracted from swine hepatocytes, using the pair of primers of XOR1 and XOR2, respectively. The two cDNA fragments were then inserted into the pMD 18-T vector to form the plasmids of pMD18-T-XOR1 and pMD18-T-XOR2 respectively, and the two plasmids were digested with *Mun* I and *Sal* I followed by ligating the two digested products, XOR1′ and pMD18-T-XOR2′, to form the plasmid of pMD18-T-XOR.

Nishino et al. [32] has reported that the recombinant rat liver XOR could be partially expressed in the baculovirus-Sf9 insect cell system in active form of native dimmer. Therefore, the insect cell system was employed for heterogeneous expression of porcine XOR and site-directed mutagenesis study. PCR amplification of the complete cDNA, using pMD18-T-XOR as template, was conducted employing the forward (Fwd) and reverse (Rev) XOR primers. Then the product was digested with *Sal* I and *Kpn* I, and inserted in frame into *Sal* I -*Kpn* I sites of pFastBac HTb vector to form the recombinant plasmid of pFastBac HTb-XOR with an N-terminal 6-His fusion. The mutants were constructed by one step site-directed mutagenesis as previously described [33]. All the constructed plasmids were sequenced and confirmed by GenScript (Nanjing, China).

### Expression of the porcine XOR and its mutants

The recombinant vector of pFastBac HTb-XOR and the empty vector of pFastBac HTb were respectively transformed into *E. coli* DH10Bac cells containing the baculovirus genome, and positive clones were selected as white colonies, using 5-bromo-4-chloro-3-indolyl-β-D-galactopyranoside (X-gal) as substrate. The recombinant baculovirus DNA was isolated from DH10Bac white colonies and then transfected into Sf9 cells by Insect GeneJuice® Transfection Reagent (Merck, Darmstadt, Germany). The medium containing viruses was collected three to four days post transfection and preserved as virus stock.

For each batch of enzyme preparation, Sf9 cells were cultured to 2×10^7^ cells/liter at 27°C in flasks in Grace's insect medium containing 10% fetal bovine serum (FBS) (Invitrogen, Carlsbad, CA, USA), 10 µM riboflavin, 10 µM guanosine, 80 µM FeSO_4_ and 0.5 mM Na_2_MoO_4_. Then the cells were infected with recombinant virus at concentration six times higher than that under expression conditions. The cells were harvested and homogenized as previously described [32], and the Sf9 cytosolic proteins containing the recombinant enzymes were obtained following the procedural instruction of Membrane and Cytosol Protein Extraction Kit (Beyotime, Jiangsu, China).

### SDS-PAGE and protein quantification

The SDS-PAGE was carried out with glycine-Tris buffer system [34]. The gel was stained with Coomassie brilliant blue G-250. The amount of cytosolic proteins was determined according to the Bradford method [35] with bovine serum albumin (BSA) as standard. The amounts of target proteins (XOR) were estimated by comparing band intensities with BSA on the gel using GeneTools gel imaging software (Syngene, Los Altos, CA, USA).

### Immunoblot analysis

Proteins separated by SDS-PAGE were electrophoretically transferred onto a nitrocellulose membrane. The blotted membrane was blocked with skim milk. The membrane was then incubated with the monoclonal 6-His antibody (Beyotime) diluted 1∶2000 in TBS containing 0.05% (v/v) Tween-20 (TBST) at room temperature for 1 h. After washing three times with TBST, the blotted membrane was treated with horseradish peroxidase (HRP)-conjugated goat anti-mouse IgG secondary antibody (Beyotime) diluted 1∶500 in TBST at room temperature for 1 h, followed by washing three times with TBST. The immunoreactive proteins were identified using 3,3′-Diaminobenzidine Tertrahydrochloride Horseradish Peroxidase Color Development Kit (Beyotime).

### Homology model and protein-ligand interaction study

For homology structure modeling, protein sequence alignment was performed by Clustal X software [36]. A three dimensional (3D) homology model of porcine XOR was constructed by Protein Modeling module of Discovery Studio 2.1 (DS 2.1) software (Accelrys, San Diego, USA) available from http://www.accelrys.com/. To choose a template structure appropriate for modeling, the protein sequence of porcine XOR was screened against PDB structure database (http://www.rcsb.org/pdb/home/home.do). From a series of templates, the 3D structure of bovine milk XOR (PDB ID: 1FIQ), which shows the highest sequence identity (91%) to that of porcine XOR, was selected as the modeling template. The protein model was generated, refined and validated as previously described [37].

For protein-ligand interaction study, the cyadox structure was generated with ChemDraw software (CambridgeSoft, Waltham, MA, USA), and optimized using “Prepare Ligands” in the DS 2.1 for docking. Cyadox as the ligand molecule was docked into the refined protein model of porcine XOR using LibDock and Flexible Docking tool of DS 2.1, which employs a genetic algorithm and allows full ligand flexibility and partial protein flexibility. The most suitable docking mode with highest score and lowest energy conformation was finally selected. The binding sites were defined as amino acid residues in distances less than 4 Å to cyadox.

### Enzymatic activity assay

The Sf9 cytosolic proteins containing the recombinant enzymes were used as the enzyme sources, and the enzymatic activities were measured using cyadox and xanthine as substrate. Cytosolic proteins of Sf9 cells transfected with recombinant baculovirus DNA containing the empty vector of pFastBac HTb were defined as XOR-free enzyme source and were used as negative control. For oxidation of xanthine, the reaction mixture contained 0.1 µg/ml porcine XOR or its mutants, and 50∼1000 µM xanthine in 50 mM Tris-HCl buffer (pH 7.4) in a final volume of 200 µl. The reaction was initiated by incubation at 37°C for 20 min. For reduction of cyadox, 25∼400 µM of cyadox were added in the reaction mixture containing 0.1 µg/ml porcine XOR or its mutants and 100 µM of xanthine in 50 mM Tris-HCl buffer (pH 7.4) in a final volume of 200 µl, and then the mixtures were incubated at 37°C for 60 min and were kept in anaerobic condition (99.999% N_2_). The reactions were stopped by boiling for 12 min followed by centrifugation at 12,000 g and 4°C for 12 min. The supernatants were then subjected to high-performance liquid chromatography (HPLC) for analysis.

HPLC was performed using Waters 2695 HPLC System equipped with the Waters 2687 Dualλ absorbance detector. To separate cyadox and its reduced metabolite, cyadox-1-monoxide, 40 µL of sample was injected into Agilent Eclipse™ XDB-C18 column (250 mm×4.6 mm, 5 µm). The mobile phase consisted of solvent A (0.5% formic acid) and solvent B (acetonitrile). The gradient elution program was set as follows: 0–5 min, 15% solvent B; 5–15 min, 15% to 45% solvent B; 15–15.1 min, 45% to 15% solvent B; 15.1–20 min, 15% solvent B. The wavelength was set at 305 nm. For the separation of xanthine and its metabolite, uric acid, the isocratic elution program was as follows: 5% solvent B. The wavelength was set at 265 nm. Quantitative analysis of cyadox-1-monoxide or uric acid was carried out according to the peak area.

Kinetic parameters for the biotransformation of xanthine and cyadox were calculated by fitting data into the Michaelis-Menten equation, v = V_max_ [S]/(K_m_+[S]), transformed as double reciprocal equation, 1/v = 1/V_max_+K_m_/V_max_ [S]. The intrinsic clearance (CL_int_) is the proportionality constant between rate of metabolism (v) and the drug substrate concentration at the enzyme site ([S]). When K_m_>>[S], CL_int_ = v/[S] = V_max_/K_m_.

### Statistical analysis

Descriptive statistical parameters such as mean and standard deviation were calculated. Statistical analysis of the data was performed using Microsoft Excel 2003.

## Results

### Cloning, sequencing and homology analysis of porcine XOR

After cloning and sequencing, the sequence of porcine liver XOR cDNA was submitted to GenBank (accession number: JN896312.1). The cDNA consists of 4005 bp nucleotides, translated as 1334 aa peptide. The entire protein sequence of porcine XOR was aligned with those of reported mammalian XORs by Clustal X [36] (Figure S1). All these mammalian XORs showed similar length and conserved amino acid sequences (80% or more overall identity). The identities of the protein sequence of porcine (*Sus scrofa*) XOR (protein ID: AEW10559) with those of bovine (*Bos taurus*) (NP_776397) [30], cat (*Felis catus*) (NP_001009217) [31], human (*Homo sapiens*) (NP_000370) [29], mouse (*Mus musculus*) (NP_035853) [28] and rat (*Rattus norvegicus*) (NP_058850) [27] were 91%, 87%, 89%, 85% and 86%, respectively. In porcine XOR, Glu804 and Arg882 (swine numbering) in the active sites of Mo-pt center, and loop 424–434 (swine numbering) involved in the conversion from XDH to XO are universally conserved when compared with XORs from other mammals (Figure S1).

### Homology model of porcine XOR and docking of cyadox

**Figure 2:**
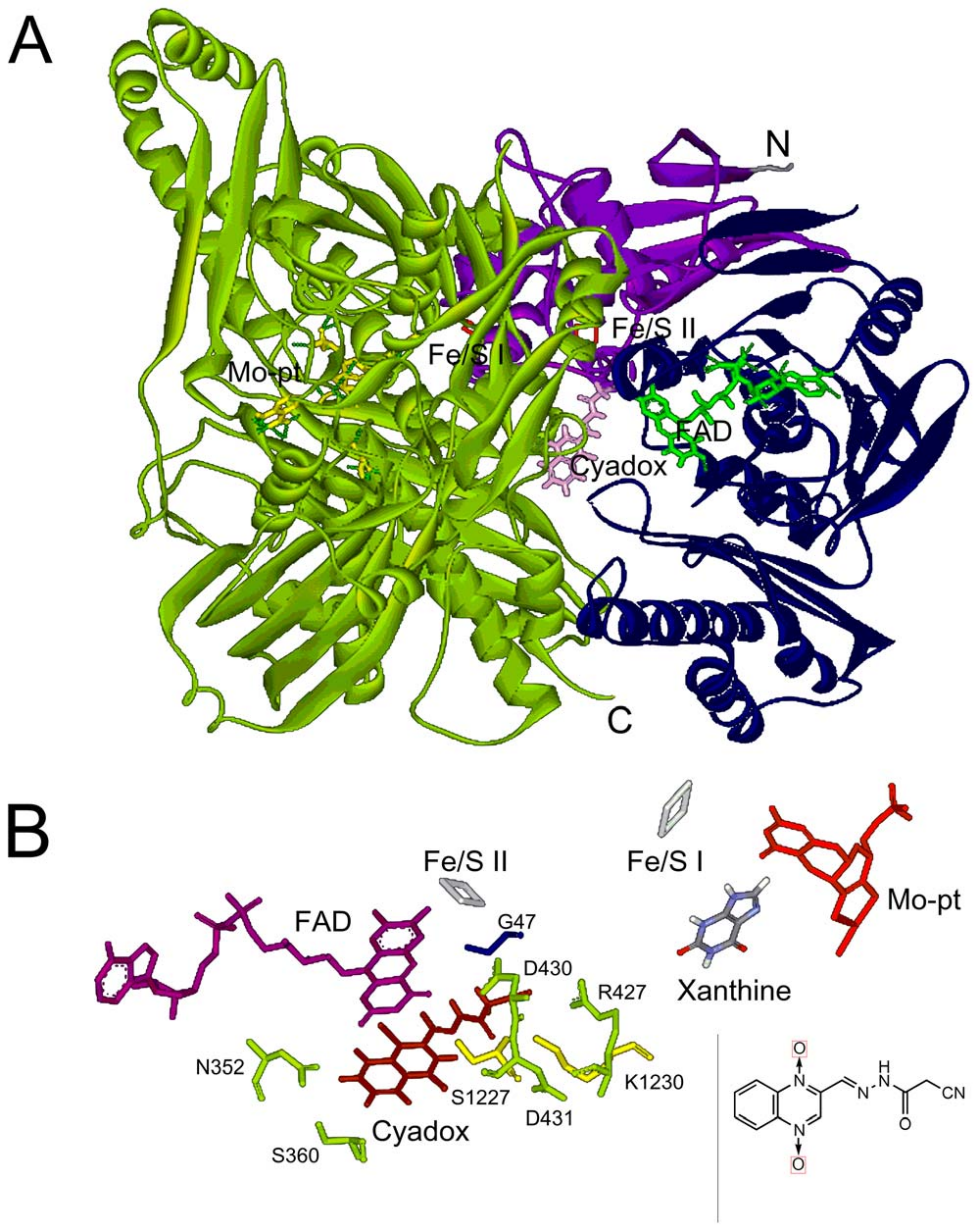
Structural overview (A) and the binding pocket (B) of porcine XOR docked with cyadox. (A) From N to C terminus, iron-sulfur centers domain (purple), FAD domain (dark blue), and molybdopterin (Mo-pt) domain (green) are presented in the 3D structure of porcine XOR model, and the Mo-pt cofactor (yellow), the two iron-sulfur centers (Fe/S I and Fe/S II, red), and the FAD cofactor (bright green) are also included. Cyadox (pink) is docked into the porcine XOR model. (B) Side chains of eight key amino acid residues, Gly47 (blue, in iron-sulfur centers domain), Asn352, Ser360, Arg427, Asp430 and Asp431 (green, in FAD domain), Ser1227 and Lys1230 (yellow, in Mo-pt domain), were screened in a distance of less than 4Å with cyadox by molecular docking. The Mo-pt cofactor (red), the Fe/S I and Fe/S II (grey), the FAD cofactor (purple), cyadox (dark red) and xanthine (blue and grey) are also included.

The X-ray crystal structure of bovine milk XOR (PDB ID: 1FIQ) was chosen as template since it exhibited highest identity (91%) of protein sequence to that of porcine XOR. Among the five models generated by the MODELER program in DS 2.1 software, the model with the lowest probability density function (PDF) total energy and discrete optimized potential energy (DOPE) score was selected and further refined by energy minimization (Figure 2A). The quality of the refined model was assessed by PROCHECK Ramachandran plot, which evaluates the stereochemical quality of a protein structure by analyzing residue-by-residue geometry and overall structural geometry [38]. The refined model demonstrated that 91.5% of the residues were in the most favored regions, 5.7% in additional allowed regions, and 2.8% in generously allowed regions. The refined model yielded Verify Score of 616.831, which was well within the range of high quality. Overall, the refined homology model of porcine XOR monomer is all well within the acceptable range.

As shown in Figure 2A, the porcine XOR monomer model, consisting of three domains, iron-sulfur center domain (residues 3–165), FAD domain (residues 227–532), and Mo-pt domain (residues 592–1333) from N to C terminus, was similar to the structures of other XORs reported to date. The Mo-pt cofactor, the two iron-sulfur centers (Fe/S I and Fe/S II) and the FAD cofactor formed an electron transfer chain, and the distances were less than 15 Å between adjacent cofactors. A long narrow channel existed between Mo-pt domain and the other two domains, and this channel was suggested to be involved in the substrate entering and binding [39]. The model of porcine XOR docked with cyadox demonstrated that the ligand was in a deep hydrophobic pocket, surrounded by the side chains of Gly47, Asn352, Ser360, Arg427, Asp430, Asp431, Ser1227 and Lys1230 (Figure 2B). All these amino acid residues were located in flexible loops and appeared in distances of less than 4Å to cyadox. These residues might be closely related to the catalytic process of porcine XOR in the metabolism of cyadox, and were investigated by the following mutagenesis study.

### Expression of porcine XOR and its mutants in Sf9 insect cells

**Figure 3:**
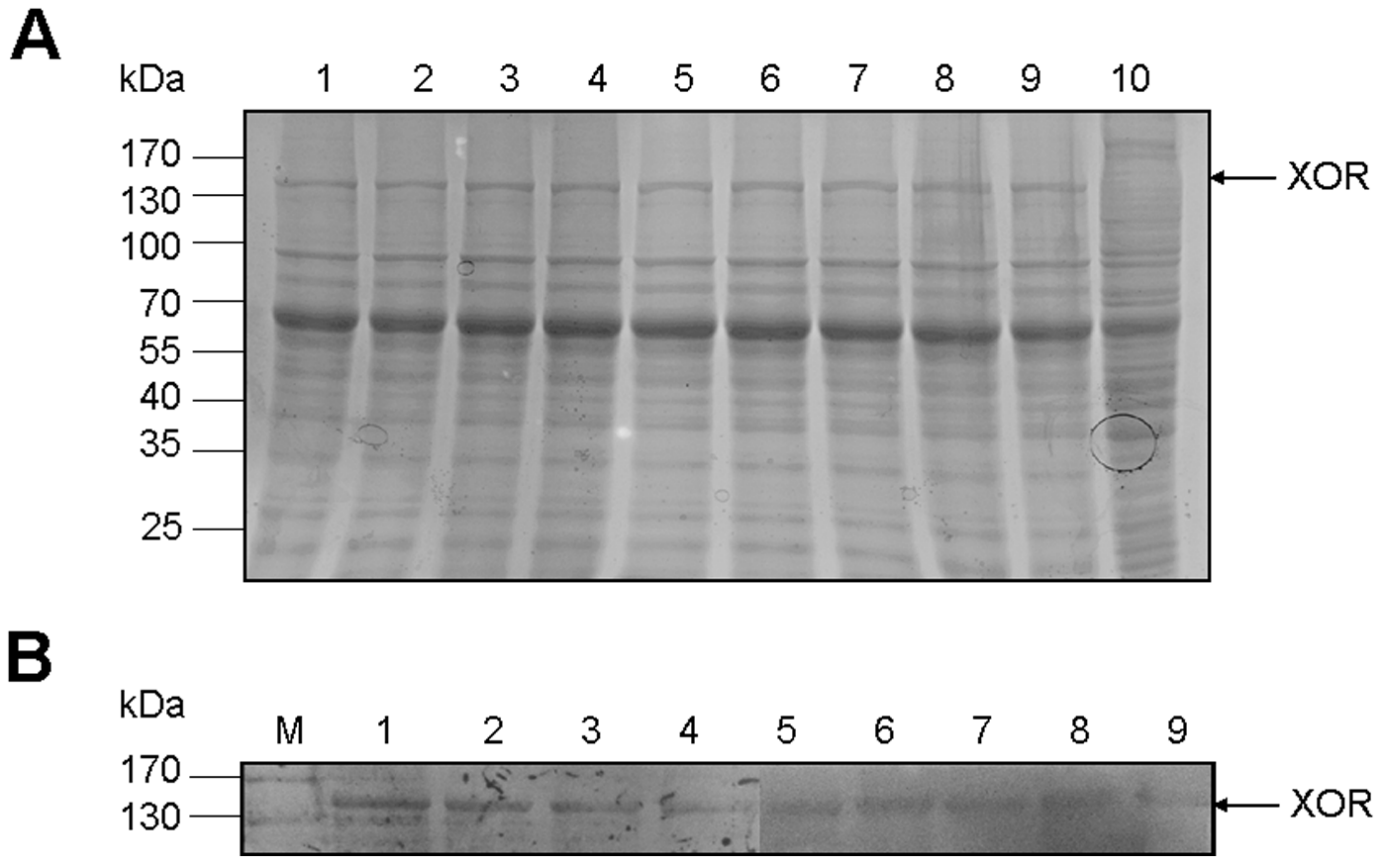
SDS-PAGE (A) and western blot (B) analysis of porcine XOR and its mutants expressed in Sf9 insect cells. (A) Lanes 1–10 were loaded with cytosolic proteins of Sf9 insect cells expressing porcine XOR mutants of G47A, N352A, S360P, R427E, D430H, D431A, S1227A, K1230A, wild type XOR and empty vector control, respectively. (B) Lanes 1–9 were loaded with proteins as described above. Monoclonal 6-His antibody was used as primary antibody to immunoblot the expressed XOR proteins as described in materials and methods. Arrows indicate the position of XOR. M, protein molecular mass standard.

Porcine XOR was expressed in Sf9 insect cells and approximate 2 mg of XOR was harvested per 10^7^ cells. Compared with the cytosolic proteins form cells harboring the empty vector (Figure 3A, lane 10), the porcine XOR, appearing as a 150 kDa peptide, was expressed in the cytosol of Sf9 insect cells (Figure 3, lane 9). This molecular weight of 150 kDa is in consistence with the fact that porcine XOR contains 1334 aa with a molecular mass of 146974.9 Da.

In order to investigate the functions of the eight amino acid residues screened by molecular docking, the residues were site-directed mutated. The basic residue Arg427 was replaced with glutamic acid, while the acidic residue Asp430 was replaced with basic residue histidine. The polar residue Ser360 was replaced with non-polar residue proline, and Gly47, Asn352, Asp431, Ser1227 and Lys1230 were replaced with non-polar residue alanine. The expressions of these eight mutants were confirmed by SDS-PAGE and immunoblot analysis (Figure 3, lane 1–8).

### The enzymatic activity of porcine XOR in the metabolism of xanthine and cyadox

As shown in Figure S2B, nearly no uric acid was generated when xanthine was incubated with Sf9 cell cytosol free of porcine XOR. When xanthine was incubated with the cytosol containing recombinant porcine XOR, the uric acid was generated (Figure S2C), demonstrating that the heterogeneously expressed porcine XOR was of biological activity. In the absence of xanthine, cyadox could not be metabolized to monoxide when incubated with the cytosol containing porcine XOR (Figure S3C), while in the presence of xanthine, the cyadox-1-monoxide was produced (Figure S3D), indicating that cyadox required an electron donated by xanthine to be reduced to produce cyadox-1-monoxide. The cytosol without recombinant porcine XOR could not metabolize cyadox (Figure S3B).

### Enzyme kinetic analysis of recombinant porcine XOR and its mutants

First, the kinetic parameters of porcine XOR and eight mutants were compared, in the oxidation of their physiological substrate, xanthine. As shown in Table 2, the CL_int_ value of D431A (392.31 mL/mg XOR/min) was higher than that of the wild type XOR (356.85 mL/mg XOR/min). S360P (44.05 mL/mg XOR/min), S1227A (77.54 mL/mg XOR/min), and K1230A (102.63 mL/mg XOR/min) exhibited much lower CL_int_ values, and R427E showed a slight lower CL_int_ values compared with the wild type. The K_m_ values of S360P (64.28 µM), S1227A (50.80 µM) and K1230A (39.25 µM) were much higher than that of wild type (21.69 µM), indicating these mutants had much weaker substrate affinity.

**Table 2:** Enzyme kinetic parameters of porcine XOR and its mutants in the oxidation of xanthine.

Enzymes	V_max_ (µmol/min/mg XOR)	K_m_ (µM)	CL_int_ (mL/mg XOR/min)
Wild type	7.74±0.03	21.69±2.27	356.85±8.13
G47A	7.91±0.01**	24.13±1.71	328.66±22.86
N352A	7.69±0.03	22.70±0.65	339.08±8.13
S360P	2.83±0.03**	64.28±1.00**	44.05±0.27**
R427E	7.75±0.04	23.24±0.90	333.33±5.12*
D430H	7.47±0.03**	21.30±0.59	350.99±8.71
D431A	8.52±0.05**	21.72±0.74	392.31±10.88*
S1227A	3.94±0.06**	50.80±3.04**	77.54±1.70**
K1230A	4.02±0.03**	39.25±1.73**	102.63±3.72**

Note: The values are expressed as means±standard deviations of the results of three independent experiments.

**(*p*<0.01) and *(*p*<0.05) indicate statistically significant difference in enzyme kinetic parameters between the wild type and each mutant.

Using cyadox as the terminal electron acceptor, the kinetic parameters of porcine XOR and its mutants were analyzed based on the yield of cyadox-1-monoxide (Table 3). Although cyadox could be metabolized by porcine XOR to yield both cyadox-1-monoxide and cyadox-4-monoxide, it could also be non-enzymatically reduced to cyadox-4-monoxide mediated by heme groups of catalase in the cytosol [20]. Furthermore, as shown in Figure S3, cyadox-1-monoxide was the main product of cyadox catalyzed by porcine XOR. Therefore, only the production of cyadox-1-monoxide was monitored here to gain the kinetic parameters. In consistence with the changes of kinetic parameters in the oxidation of xanthine, the CL_int_ values of S360P (8.3 mL/mg XOR/min), S1227A (5.5 mL/mg XOR/min), K1230A (5.4 mL/mg XOR/min) were much lower than that of the wild type (23.17 mL/mg XOR/min) because of the lower V_max_ and higher K_m_ values. Besides, D431A (24.77 mL/mg XOR/min) showed higher CL_int_ value, and G47A exhibited only a slight higher V_max_, compared with the wild type. In contrast, the CL_int_ value of R427E (68.67 mL/mg XOR/min) was much higher than that of the wild type, and the K_m_ (20.86 µM) was much lower than that of wild type (58.20 µM), demonstrating R427E had a strong substrate affinity. Meanwhile, D430H exhibited both lower V_max_ and CL_int_ values than those of the wild type.

**Table 3:** Enzyme kinetic parameters of porcine XOR and its mutants in the reduction of cyadox.

Enzymes	V_max_ (µmol/min/mg XOR)	K_m_ (µM)	CL_int_ (mL/mg XOR/min)
Wild type	1.34±0.05	58.20±5.76	23.17±1.48
G47A	1.41±0.03*	65.68±4.42	21.45±1.05
N352A	1.29±0.18	60.10±12.59	21.68±1.68
S360P	0.78±0.04**	93.53±8.15**	8.33±0.36**
R427E	1.43±0.05	20.86±0.90**	68.67±5.34**
D430H	0.98±0.03**	64.68±3.52	15.22±0.49**
D431A	1.52±0.01**	61.29±0.53*	24.77±0.25*
S1227A	0.81±0.05**	148.20±14.21**	5.46±0.18**
K1230A	0.88±0.08**	162.89±15.50**	5.40±0.10**

Note: The values are expressed as means±standard deviations of the results of three independent experiments.

**(*p*<0.01) and *(*p*<0.05) indicate statistically significant difference in enzyme kinetic parameters between the wild type and each mutant.

## Discussion

The present study has demonstrated that the porcine XOR was expressed in Sf9 cells with XDH/XO activity. The enzyme possesses common redox active cofactors, forming an electron transfer pathway terminated by a flavin cofactor. When cyadox, an exogenous electron acceptor, was docked into porcine XOR, eight amino acid residues, which are conservative among the XORs from other mammalian species, were screened out as the potential cyadox-binding sites. The side chain of cyadox oriented toward Gly47 located in iron-sulfur center domain, Arg427 in the FAD domain, and Ser1227 and Lys1230 in the Mo-pt domain. Meanwhile, the benzene ring of quinoxaline heterocycle faced toward Asn352 and Ser360 in the FAD domain, and the pyrazine ring was located near Asp430 and Asp431 in the FAD domain. It has been shown that one electron reduction of cyadox occurs at one of the two N→O groups on the pyrazine ring [11]. However, our docking result demonstrated that the pyrazine ring of cyadox faced at FAD center, and this was different form the studies about the reduction of nicotinamide *N*-oxide and nitrite by XOR which both suggested that the reactions involved the abstraction of an oxygen atom and occurred at the molybdenum centre [40], [41]. This is probably due to the influence of the large side chain of cyadox upon binding to the enzyme.

In order to obtain the enzyme kinetic parameters of porcine XOR in the reduction of cyadox, the production of one of the reductive metabolites, cyadox-1-monoxide, was monitored, as to exclude the influence of the fact that cyadox could also be reduced to cyadox-4-monoxide by non-enzymatic reduction mediated by heme groups of catalase in the cytosol [20]. Since oxygen could oxidize cyadox-1-monoxide back to its dioxide form [21], the reaction was kept under anaerobic condition. G47A, S360P, D431A, S1227A, and K1230A displayed differential enzyme kinetic parameters compared with those of the wide type (Table 3). However, these changes were similar to those in the oxidation of xanthine (Table 2), indicating that these mutations affect the metabolism of xanthine which serves as the electron donor, resulting in impairment of the subsequent electron transfer that is critical for the reduction of cyadox as the electron acceptor. Among these residues, S360P, S1227A, K1230A exhibited significantly lower CL_int_ values for xanthine because of the lower V_max_ and higher K_m_ values. Since Ser1227 and Lys1230 are located in the Mo-pt domain, which contains the binding and active sites of XOR for xanthine, the mutations of these residues may affect the binding and catalyzing of xanthine by XOR. Since Ser360 is located at the *N*-terminal of α10 helix (Figure S1), and proline is a rigid amino acid which can destroy the formation of α-helix, this mutation presumably results in the whole structural change of porcine XOR.

Different from the alterations of kinetic parameters in the oxidation of xanthine (Table 2), mutation of Arg427 to glutamic acid displayed a higher CL_int_ and a much lower K_m_ values than those of the wild type in the reduction of cyadox (Table 3), suggesting that R427E had a strong substrate binding affinity for cyadox. Arg427 is located in the 424–434 loop (swine numbering, equivalent to 423–433 loop in bovine XOR, Figure S1), the orientation of which is important for the transition from XDH to XO [10]. The two forms of this enzyme prefer different ultimate substrate (NAD^+^ for XDH and O_2_ for XO) for electron transfer, and possess different redox potentials for the flavin semiquinone/hydroquinone pair (E_sq/hq_) [42], [43]. It is reported that Arg426 (bovine numbering, equal to Arg427 in porcine XOR) is one of the key residues that differentiate the E_sq/hq_ for XDH and XO significantly [2]. We suppose that the charge change of R427E may influence the substrate specificity and binding of the enzyme. More mutants need to be made to elucidate the exact role of Arg427 in the reduction of cyadox. Since the mutagenic activity of QdNOs is mainly caused by the existence of *N*-oxide groups in QdNO structures [11], the greater CL_int_ of R427E for cyadox is of favorable applications in the detoxification of cyadox in animal feed.

Meanwhile, D430H exhibited a lower CL_int_ due to the lower V_max_ values, indicating a deficiency in reduction of cyadox (Table 3). Asp430 is also located in the 424–434 loop. In order to activate an electron transfer from Fe/S II to flavin, the enzyme needs an upshift of E_sq/hq_ to make electron transfer from Fe/S II to the flavin isoenergetic [44]. Recent study reported that no large conformational change was observed in response to NAD^+^ binding at bovine XDH. Instead, the positive charge of the NAD^+^ ring, deprotonation of Asp429 (equivalent to Asp430 in porcine XOR), and capping of the bulk surface of the flavin by the NAD^+^ molecule all contributed to altering E_sq/hq_ upon NAD^+^ binding to XDH [2]. Based on this data, we speculate that D430H of porcine XOR harms the deprotonation of Asp430 and influence the E_sq/hq_ alteration which is needed for the electron transfer to the terminal electron acceptor, cyadox. In addition, the K_m_ of K1230A was nearly three times to that of wild type in the reduction of cyadox, while only two-fold upshift of K_m_ was detected in the oxidation of xanthine. It was reported that although Lys1228 (bovine numbering, equal to Lys1230 in porcine XOR) did not belong to 423–433 loop (bovine numbering), it still significantly influenced the E_sq/hq_ for both XDH and XO [2].

In conclusion, our work for the first time cloned the cDNA of XOR from porcine liver and obtained functionally active recombinant XOR expressed in Sf9 insect cells. Using molecular docking combined with site-directed mutagenesis techniques, it is demonstrated that Arg427 is related to the substrate binding of porcine XOR to cyadox. Asp430 probably plays a role in the electron transfer to cyadox. Besides, Gly47, Ser360, Asp431, Ser1227 and Lys1230 participate in the electron-donating process of xanthine, whose obstruction hinders the fulfillment of *N*-oxide reduction of cyadox. Although molecular docking is a predictive method, it still verifies the result of mutation and kinetic analysis of porcine XOR for cyadox catabolism in our study. To get a better understanding of the catalytic mechanism underlying the reductive activation of QdNOs by porcine XOR, further study will be devoted into the crystal structure analysis of porcine XOR in complex with QdNO substrate.

## Supporting Information

Figure S1 **Amino acid sequence alignment of porcine XOR with other five mammalian XORs.** Totally conserved residues of XORs in all 6 mammalian species are shown by asterisks (*), partially conserved residues are shown by dots (.or:). The second structures were predicted by PSIPRED V3.0 software (http://bioinf.cs.ucl.ac.uk/psipred/). The α-helices are shown in underlined letters, and the β-strands in boldface letters. The universally conserved Glu and Arg in the active sites of Mo-pt center are marked with filled circles (•). The eight key amino acid residues, Gly47, Asn352, Ser360, Arg427, Asp430, Asp431, Ser1227 and Lys1230 (swine numbering), generated by molecular docking, are indicated as numbered underlined letters in bold.
(DOC)

Figure S2 HPLC chromatograms of the metabolite of xathine catalyzed by recombinant porcine XOR. (A) Uric standard. (B) Xanthine incubated with Sf9 cell cytosol free of recombinant porcine XOR at 37°C for 20 min. (C) Xanthine incubated with Sf9 cell cytosol containing recombinant porcine XOR at 37°C for 20 min.
(PPT)

Figure S3 HPLC chromatograms of the metabolite of cyadox catalyzed by recombinant porcine XOR. (A) Cyadox-1-monoxide standard. (B) Cyadox incubated with Sf9 cell cytosol free of recombinant porcine XOR with xanthine at 37°C for 60 min. (C) Cyadox incubated with Sf9 cell cytosol containing recombinant procine XOR in the absence of xanthine at 37°C for 60 min. (D) Cyadox incubated with Sf9 cell cytosol containing recombinant porcine XOR in the presence of xanthine at 37°C for 60 min.
(PPT)
