# Supplementary material for: Mechanism of Porcine Liver Xanthine Oxidoreductase Mediated N-Oxide Reduction of Cyadox as Revealed by Docking and Mutagenesis Studies
Source: PLoS One. 2013 Sep 9;8(9):e73912. doi: 10.1371/journal.pone.0073912 (PMC3767608; doi:10.1371/journal.pone.0073912)
Supplement: Figure S1 — Amino acid sequence alignment of porcine XOR with other five mammalian XORs. Totally conserved residues of XORs in all 6 mammalian species are shown by asterisks (*), partially conserved residues are shown by dots (.or:). The second structures were predicted by PSIPRED V3.0 software (http://bioinf.cs.ucl.ac.uk/psipred/). The α-helices are shown in underlined letters, and the β-strands in boldface letters. The universally conserved Glu and Arg in the active sites of Mo-pt center are marked with filled circles (•). The eight key amino acid residues, Gly47, Asn352, Ser360, Arg427, Asp430, Asp431, Ser1227 and Lys1230 (swine numbering), generated by molecular docking, are indicated as numbered underlined letters in bold. (DOC) [file pone.0073912.s001.doc]

**Supporting Information: Figure S1**

β1 β2 α1 47 β3

swine 1 ---MTAD**ELVFFV**NG**KKVVE**KNADPETTLLAYLRRKLGLRGTKLGCGEG**G**CGA**CTVMFS**K 57

bovine 1 ---MTAD**ELVFFV**NG**KKVVE**KNADPETTLLAYLRRKLGLRGTKLGCGEGGCG**ACTVML**SK 57

cat 1 ---MTAD**ELVFFV**NG**KKVVE**KNADPETTLLAYLRRKLGLSGTKLGCGEGGCGA**CTVML**SK 57

human 1 ---MTAD**KLVFFV**NG**RKVVE**KNADPETTLLAYLRRKLGLSGTKLGCGEGGCG**ACTVMLSK** 57

mouse 1 MTRTTVD**ELVFFV**NG**KKVV**EKNADPETTLLVYLRRKLGLCGTKLGCGEGGCG**ACTVMISK** 60

rat 1 ---MTAD**ELVFFV**NG**KKVVE**KNADPETTLLVYLRRKLGLCGTKLGCGEGGCGA**CTVMISK** 57

*.*:*******:**************.******** *****************:**

β4 β5 α2

swine 58 YDRLQDK**IVHFS**ANACLAPICSLHHV**AVTTV**EGIGSTKTRLHPVQERIAKSHGSQCGFCT 117

bovine 58 YDRLQDK**IIHFS**ANACLAPICTLHHV**AVTTVE**GIGSTKTRLHPVQERIAKSHGSQCGFCT 117

cat 58 YDRFQNK**IVHFSA**NACLAPICSLHHV**AVTTVE**GIGSTKSRLHPVQERIAKSHGSQCGFCT 117

human 58 YDRLQN**KIVHFSA**NACLAPICSLHHV**AVTTVE**GIGSTKTRLHPVQERIAKSHGSQCGFCT 117

mouse 60 **Y**DRLQN**KIVHFSV**NACLTPICSLHHV**AVTTVE**GIGNTK-KLHPVQERIAKSHGSQCGFCT 119

rat 58 **Y**DRLQNK**IVHFSV**NACLAPICSLHHV**AVTTVE**GIGNTQ-KLHPVQERIARSHGSQCGFCT 116

***:*:**:***.****:***:*************.*: :*********:**********

α3 α4 α5

swine 118 PGIVMSMYTLLRNQPEPTVEEIEDAFQGNLCRCTGYRPILQGFRTFAKDGGCCGGSGDTP 177

bovine 118 PGIVMSMYTLLRNQPEPTVEEIEDAFQGNLCRCTGYRPILQGFRTFAKNGGCCGGNGNNP 177

cat 118 PGIVMSMYTLLRNQPEPTIEEIEDAFQGNLCRCTGYRPILQGFRTFARDGGCCGGSGNDL 177

human 118 PGIVMSMYTLLRNQPEPTMEEIENAFQGNLCRCTGYRPILQGFRTFARDGGCCGGDGNNP 177

mouse 120 PGIVMSMYTLLRNKPEPTVEEIENAFQGNLCRCTGYRPILQGFRTFAKDGGCCGGSGNNP 179

rat 117 PGIVMSMYTLLRNQPEPTVEEIENAFQGNLCRCTGYRPILQGFRTFAKDGGCCGGSGNNP 176

*************:****:****:***********************::******.*:

α6 β6 β7

swine 178 NCCLNQKKDHKQVTLSPSLFNAEEFMPLDPTQEPIFPPELLRLKDTPQKQ**LRFE**GER**VTW** 237

bovine 178 NCCMNQKKDHT-VTLSPSLFNPEEFMPLDPTQEPIFPPELLRLKDVPPKQ**LRFE**GER**VTW** 236

cat 178 NCCMNQKTDHK-ITLSPSLFNPEEFTPLDPTQEPIFPPELLRLKDTPQKQ**LRFE**GER**VTW** 236

human 178 NCCMNQKKDHS-VSLSPSLFKPEEFTPLDPTQEPIFPPELLRLKDTPRKQ**LRFE**GER**VTW** 236

mouse 180 NCCMSQTKDQT-IAPSSSLFNPEDFKPLDPTQEPIFPPELLRLKDTPRKT**LRFE**GER**VTW** 238

rat 177 NCCMNQTKDQT-VSLSPSLFNPEDFKPLDPTQEPIFPPELLRLKDTPQKK**LRFE**GERV**TW** 235

***:.*..*:. :: *.***:.*:* *******************.* * **********

α7 β8 β9 β10 β11

swine 238 **IQ**ASTLKELLDLKAQHPEA**KLVV**GNTEP**GVEMKF**KNRLFP**VIIC**PAWIPELNS**VEQ**GLEG 297

bovine 237 **IQ**ASTLKELLDLKAQHPEA**KLVV**GNTEI**GIEMKF**KNQLFP**MIIC**PAWIPELNA**VEH**GPEG 296

cat 237 **IQA**STLQELLDLKAQDPEAK**LVV**GNTEIG**IEMKFK**NMLFP**KMV**CPAWIPEP—**VEH**GPEG 294

human 237 **IQ**ASTLKELLDLKAQHPDA**KLVV**GNTEI**GIEMKFK**NMLFP**MIVC**PAWIPELNS**VEH**GPD**G** 296

mouse 239 **IQV**STMEELLDLKAQHPDA**KLVV**GNTE**IGIEMKF**KNMLFP**LIIC**PAWILELTS**VAH**GPEG 298

rat 236 **IQ**ASTMEELLDLKAQHPDA**KLVV**GNTE**IGIEMKF**KNMLFP**LIVC**PAWIPELNS**VVH**GPEG 295

**.**::********.*:********* *:****** *** ::***** * * :* :*

β12 α8 α9 352 β13

swine 298 **ISF**GAACTLSAVEKTLLDAVAKLPSHKTEVFRGVLEQLRWFAGKQVKAVASIGG**NIIT**AS 357

bovine 297 **ISF**GAACALSSVEKTLLEAVAKLPTQKTEVFRGVLEQLRWFAGKQVKSVASLGGN**IIT**AS 356

cat 295 **ISFGA**SCPLSLVEKTLLDAVANLPAHQTEVFKGVLEQLRWFAGKQVKSVASIGGN**II**TAS 354

human 297 **ISF**GAACPLSIVEKTLVDAVAKLPAQKTEVFRGVLEQLRWFAGKQVKSVASVGGN**II**TAS 356

mouse 299 **ISF**GAACPLSLVESVLADAIATLPEQRTEVFRGVMEQLRWFAGKQVKSVASIGGNIITAS 358

rat 296 **ISF**GASCPLSLVESVLAEEIAKLPEQKTEVFRGVMEQLRWFAGKQVKSVASIGGN**IIT**AS 355

*****:*.** **..* : :*.** ::****:**:************:***:********

360 α10 β14 β15 β16

swine 358 PI**S**DLNPVFMASRA**KLTIVS**RGT**RRTVP**MDHTFFPSYRKTLLGPEE**ILLSIE**IPYSREGE 417

bovine 357 PISDLNPVFMASGT**KLTIVS**RGTR**RTVP**MDHTFFPSYRKTLLGPEE**ILLSIE**IPYSRE**DE** 416

cat 355 PISDLNPVFMASGA**KLTIVS**TGT**RRTV**RMDHTFFPAYRKTLLAPEE**ILLSIE**IPYSREGE 414

human 357 PISDLNPVFMASGA**KLTLVS**RGTR**RTVQ**MDHTFFPGYRKTLLSPEE**ILLSIE**IPYSREGE 416

mouse 359 PISDLNPVLMASRA**KLTLAS**RGTK**RTVW**MDHTFFPGYRRTLLSPEE**ILVSIV**IPYSRKGE 418

rat 356 PISDLNPVFMASGA**KLTLVS**RGT**RRTV**RMDHTFFPGYRKTLLRPEE**ILLSIE**IPYSKEGE 415

********:*** :***:.* **:*** *******.**:*** *****:** ****::.*

β17 427 430-1 β18 β19 α11

swine 418 FFSAFKQAS**R**RE**DD**I**AKVTCGMRVLFE**PGTT**QVKELDLCYG**GMADRT**IS**ALKTTRKQLSQ 477

bovine 417 **FFSAFK**QASRREDDI**AKVTCGMRVLFQ**PGSM**QVKELALCYG**GMADRT**IS**ALKTTQKQLSK 476

cat 415 Y**FSAFKQ**ASRREDDI**AKVTSGMRVLFN**PGTA**QVKELALCYG**GMHDRTVSALQTTRKQISN 474

human 417 Y**FSAFK**QASRREDDI**AKVTSGMRVLFK**PGTT**EVQELALCYG**GMANR**TIS**ALKTTQRQLSK 476

mouse 419 FFSAFKQASRREDDI**AKVTSGMRVLFK**PGTT**EVQELSLCFG**GMADRTVSALKTTPKQLSK 478

rat 416 F**FSAFK**QASRREDDI**AKV**TS**GMRVLFK**PGT**IEVQELSLCF**GGMADRTISALKTTPKQLSK 475

:******************.******:**: :*:** **:*** :**:***:** :*:*:

α12 α13

swine 478 FWNEKLLQDVCAGLAEELSLPPDAPGGMVEFRRTLSLSFFFRFYLTVLQKLGREDPEDKC 537

bovine 477 FWNEKLLQDVCAGLAEELSLSPDAPGGMIEFRRTLTLSFFFKFYLTVLKKLGK-DSKDKC 535

cat 475 FWNEELLQNVCAGLAEELSLAPDAPGGMVEFRRTLTLSFFFKFYLTVLQKLGIQNSKDKC 534

human 477 LWKEELLQDVCAGLAEELHLPPDAPGGMVDFRCTLTLSFFFKFYLTVLQKLGQENLEDKC 536

mouse 479 SWNEELLQDVCAGLAEELHLAPDAPGGMVEFRRTLTLSFFFKFYLTVLQKLGRADLEGMC 538

rat 476 SWNEELLQSVCAGLAEELQLAPDAPGGMVEFRRTLTLSFFFKFYLTVLQKLGRADLEDMC 535

*:*:***.********* *.*******::** **:*****:******:*** : :.

β20 α14 β21

swine 538 GKLDPTYASATWLFHKDPPANVQL**FQE**VPKGQSEEDMVGRPLPHLAAALQAS**GEAVY**CDD 597

bovine 536 GKLDPTYTSATLLFQKHPPANIQLF**QE**VPNGQSKEDTVGRPLPHLAAAMQAS**GEAV**YCDD 595

cat 535 GKLDPTHASATLLFQKDPPANVQLFQEVPKGQCEEDMVGRPLPHLAAAMQAS**GEAV**YCDD 594

human 537 GKLDPTFASATLLFQKDPPADVQL**FQE**VPKGQSEEDMVGRPLPHLAADMQA**SGEAV**YCDD 596

mouse 539 GKLDPTFASATLLFQKDPPANVQL**FQE**VPKGQSEEDMVGRPMPHLAADMQA**SGEAVY**CDD 598

rat 536 GKLDPTFASATLLFQKDPPANVQL**FQE**VPKDQSEEDMVGRPLPHLAANMQA**SGEAVY**CDD 595

******.:*** **:*.***::*******:.*.:** ****:***** :***********

β22 β23 α15 β24 β25

swine 598 IPCYENE**LFLRLVT**STRAH**AKIKSID**ISEAQKVPG**FVCFLS**ADDIPGSNEIGIFKD**ETVF** 657

bovine 596 IPRYENE**LFLRLVT**STRAH**AKIKSID**VSEAQKVPG**FVCFLS**ADDIPGSNETGLFNDE**TVF** 655

cat 595 IPRYENE**LSLRLVT**STRAH**AKIKSID**TSEAQKVPG**FVCFI**SADDVPGSNITGIGND**EMVF** 654

human 597 IPRYENE**LSLRLVT**STRAH**AKIKSID**TSEAKKVPG**FVCFIS**ADDVPGSNITGICND**ETVF** 656

mouse 599 IPRYENE**LSLRLVT**STRAH**AKIMSID**TSEAKKVPG**FVCFL**TSEDVPGSNITGIFNDE**TVF** 658

rat 596 IPRYENE**LSLRLVT**STRAH**AKITSID**TSEAKKVPG**FVCFL**TAEDVPNSNATGLFNDE**TVF** 655

** ***** ************* *** ***:********::::*:*.** *: :** **

β26 β27 α16 β28 α17

swine 658 **V**KD**KVTCV**GH**AIGAVVA**DTPEHAQRAAHGV**KVTYE**DLPAIITIEDAIKYNSFYESELKIE 717

bovine 656 AKD**TVTCV**GH**IIGAVVA**DTPEHAERAAHVV**KVTYED**LPAIITIEDAIKNNSFYGSELKIE 715

cat 655 **A**KD**KVTCI**GH**IIGAVVT**DTREHAQRAAQAV**RITYED**LPAIITIEDAIAKDSFYEPELK**IE** 714

human 657 AKD**KVTCV**GH**IIGAVVA**DTPEHTQRAAQGV**KITYE**ELPAIITIEDAIKNNSFYGPELKIE 716

mouse 659 AKDE**VTC**VGH**IIGAVVA**DTPEHAHRAARGVK**ITYE**DLPAIITIQDAIKNNSFYGPEVKIE 718

rat 656 AKD**EVTCV**GH**IIGAVVA**DTPEHAQRAARGVK**ITYE**DLPAIITIQDAINNNSFYGSEI**KIE** 715

.** ***:** *****:** **:.***: *::***:*******:*** :*** .*:***

α18 β29 β30 β31 α19

swine 718 KGDLKKGFSEADN**VVSGELYI**GGQEHFYLETH**CTIAVPK**GEAGE**MELFL**ATQNAMMAQSS 777

bovine 716 KGDLKKGFSEAD**NVVSGELYI**GGQDHFYLETH**CTIAIPK**GEEGE**MELFV**STQNAMKTQSF 775

cat 715 KGNLTKGFSEADN**IVSGELYI**GGQEHFYLETH**CTIAVPK**GEAGE**MELFV**STQNTTKTQSF 774

human 717 KGDLKKGFSEAD**NVVSGEIYI**GGQEHFYLETH**CTIAVPK**GEAGE**MELFV**STQNTMKTQSF 776

mouse 719 KGDLKKGFSEAD**NVVSGELYI**GGQEHFYLETH**CTIAVPK**GEAGE**MELFV**STQNTMKTQSF 778

rat 716 KGDLKKGFSEAD**NVVSGELYI**GG**QE**HFYLETN**CTIAVPK**GEAGE**MELFV**STQNTMKTQSF 775

**:*.********:****:*****:******:****:**** ******::***: :**

β32 ● α20 β33 α21

swine 778 VASTLGVPINR**ILVRV**KRIGGGFGGKETRGIGLTVAVALAAYKTGRP**VRCM**LDRDEDMLM 837

bovine 776 VAKMLGVPVNR**ILVRV**KRMGGGFGGKETRSTLVSVAVALAAYKTGHP**VRCM**LDRNEDMLI 835

cat 775 VANMLGVPANR**ILVRV**KRMGGGFGGKETRSTVVSTAVPLAAYKTGRP**VRC**MLDRDEDMLI 834

human 777 VAKMLGVPANR**IVVRV**KRMGGGFGGKETRSTVVSTAVALAAYKTGRP**VRCM**LDRDEDMLI 836

mouse 779 IAKMLGVPDNR**IVVRV**KRMGGGFGGKETRSTLISTAVALAAYKTGRP**VRCM**LDRDEDMLI 838

rat 776 VAKMLGVPDNR**IVVRV**KRMGGGFGGKETRSTVVSTALALAAHKTGRP**VRCM**LDRDEDMLI 835

:*. **** ***:*****:**********. ::.*:.***:***:********:****:

β34 β35 α22 ●

swine 838 TGGRHP**FLARYKVGF**MKTGK**IVALEVDHYS**NAGNSLDLSHGIMERALFHMDNSYKIPN**IR** 897

bovine 836 TGGRHP**FLARYKVGF**MKTGT**IVALEVDHYS**NAGNSRDLSHSIMERALFHMDNCYKIPN**IR** 895

cat 835 TGGRHP**FLARYKVGF**MKTGR**VVALKVEHYS**NAGNTLDLSQSIMERALFHMDNCYNIPN**IR** 894

human 837 TGGRHP**FLARYKVGF**MKTGT**VVALEVDHFS**NVGNTQDLSQSIMERALFHMDNCYKIPN**IR** 896

mouse 839 TGGRHP**FLAKYKVGF**MKTGT**IVALEVAHFS**NGGNSEDLSRSIMERAVFHMDNAYKIPN**IR** 898

rat 836 TGGRHP**FLAKYKVGF**MKTGT**VVALEVAHFSN**GGNTEDLSRSIMERALFHMDNAYKIPN**IR** 895

*********:********* :***:* *:** **: ***:.*****:*****.*:*****

β36 α23 α24

swine 898 **GTGRLCK**TNLPSNTAFRGFGGPQGMFIAEYWMSEVAVTCGLPAEEVRRKNLYKEGDLTHF 957

bovine 896 **GTGRLCKT**NLSSNTAFRGFGGPQALFIAENWMSEVAVTCGLPAEEVRWKNMYKEGDLTHF 955

cat 895 **GTGRICKT**NLPSNTAFRGFGGPQGMLIAEHWMSEVAVTCGLPAEEVRRKNMYKEGDLTHF 954

human 897 **GTGRLCKT**NLPSNTAFRGFGGPQGMLIAECWMSEVAVTCGMPAEEVRRKNLYKEGDLTHF 956

mouse 999 **GTGRICKT**NLPSNTAFRGFGGPQGMLIAEYWMSEVAVTCGLPAEEVRRKNMYKEGDLTHF 958

rat 896 **GTGRICKT**NLPSNTAFRGFGGPQGMLIAEYWMSEVAITCGLPAEEVRRKNMYKEGDLTHF 955

****:*****.************.::*** ******:***:****** **:*********

α25 α26 β37

swine 958 NQKLEGFTLPRCWDECLESSQYHARKSEVDKFNRENCW**KKRGLCIIPTKF**GVSFTIPFLN 1017

bovine 956 NQRLEGFSVPRCWDECLKSSQYYARKSEVDKFNKENCW**KKRGLCIIPTK**FGISFTVPFLN 1015

cat 955 NQKLEGFTLPRCWEECLASSQYHARKREADKFNEENCW**KKRGLSIIPTK**FGISFTVPFLN 1014

human 957 NQKLEGFTLPRCWEECLASSQYHARKSEVDKFNKENCW**KKRGLCIIPTK**FGISFTVPFLN 1016

mouse 959 NQKLEGFTLPRCWDECIASSQYQARKMEVEKFNRENCW**KKRGLCIIPTK**FGISFTLSFLN 1018

rat 956 NQKLEGFTLPRCWDECIASSQYLARKREVEKFNRENCW**KKRGLCIIPTKF**GISFTLPFLN 1015

**:****::****:**: **** *** *.:***.*********.*******:***:.***

β38 β39 α27 β40

swine 1018 Q**AGALIHV**YTDGS**VLVSH**GGTEMGQGLHTKMVQVAGRALKIPTSK**IYIS**ETSTNTVPNSS 1077

bovine 1016 Q**AGALIHV**YTDGS**VLVSH**GGTEMGQGLHTKMVQVASKALKIPISK**IYIS**ETSTNTVPNSS 1075

cat 1015 Q**AGALVHVY**TDGS**VLLTH**GGTEMGQGLHTKMVQVASRALKIPTSK**IYIS**ETSTNTVPNTS 1074

human 1017 Q**AGALLHVY**TDGS**VLLTH**GGTEMGQGLHTKMVQVASRALKIPTSK**IYIS**ETSTNTVPNTS 1076

mouse 1019 Q**GGALVHVY**TDGS**VLLTH**GGTEMGQGLHTKMVQVASRALKIPTSK**IHIT**ETSTNTVPNTS 1078

rat 1016 **QGGALVHVY**TDG**SVLLTH**GGTEMGQGLHTKMVQVASRALKIPTSK**IHIS**ETSTNTVPNTS 1075

*.***:*********::******************.:***** ***:*:*********:*

α28 α29 β41

swine 1078 PTAASVSSDIYGQAVYEACQTILKGLDPFKRKNPSGSWEDWVTAAYHDR**VSLSATGFYK**T 1137

bovine 1076 PTAASVSTDIYGQAVYEACQTILKRLEPFKKKNPDGSWEDWVMAAYQDRVS**LSTTGFYR**T 1135

cat 1075 PTAASVSTDINGQAVYEACQTILKRLEPFKKKNPSGSWEDWVTAAYLDA**VSLSATGFY**KT 1134

human 1077 PTAASVSADLNGQAVYAACQTILKRLEPYKKKNPSGSWEDWVTAAYMDT**VSLSATGFYR**T 1136

mouse 1079 PTAASASADLNGQAIYEACQTILKRLEPFKKKNPSGSWESWVMDAYTSA**VSLSATGFYK**T 1138

rat 1076 PTAASASADLNGQGVYEACQTILKRLEPFKKKKPTGPWEAWVMDAYTSA**VSLSATGFYK**T 1135

*****.*:*: **.:* ******* *:*:*:*:* *.** ** ** . ****:****:*

β42 β43 α30

swine 1138 PNLGYSFETNSGNAFHYFT**YGVACSEVEID**CLTGD**HKNLRTDIVMD**VGSSLNPAIDIGQV 1197

bovine 1136 PNLGYSFETNSGNAFHYFT**YGVACSEVEID**CLTGD**HKNLRTDIVMD**VGSSLNPAIDIGQV 1195

cat 1135 PNIGYSFETNSGNPFHYFS**YGVACSEVEID**CLTGD**HKNLRTDIVMD**VGSSLNPAIDIGQV 1194

human 1137 PNLGYSFETNSGNPFHYFS**YGVACSEVEID**CLTGD**HKNLRTDIVMD**VGSSLNPAIDIGQV 1196

mouse 1139 PNLGYSFETNSGNPFHYFS**YGVACSEVEID**CLTGD**HKNLRTDIVMD**VGSSLNPAIDIGQV 1198

rat 1136 PNLGYSFETNSGNPFHYF**SYGVACSEVEID**CLTGD**HKNLRTDIVMD**VGSSLNPAIDIGQV 1195

**:**********.****:*****************************************

α31 β44 1227 1230 β45

swine 1198 EGAFVQGLGLFTLEEL**HY**SPDGILHTRGP**S**TY**K**IPAFGSIPTEF**RVSLL**RDCPNKKAIYA 1257

bovine 1196 EGAFVQGLGLFTLEE**LHY**SPEGSLHTRGPSTYKIPAFGSIPTEF**RVSLL**RDCPNKKAIYA 1255

cat 1195 EGAFVQGLGLFTLEE**LHY**SPEGSLHTRGPSTYKIPAFGSIPSEF**RVSLL**RDCPNKKAIYA 1254

human 1197 EGAFVQGLGLFTLEE**LHY**SPEGSLHTRGPSTYKIPAFGSIPIEF**RVSLL**RDCPNKKAIYA 1256

mouse 1199 EGAFVQGLGLFTMEE**LHY**SPEGSLHTRGPSTYKIPAFGSIPIEF**RVSL**LRDCPNKRAIYA 1258

rat 1196 EGAFVQGLGLFTMEEL**HY**SPEGSLHTRGPSTYKIPAFGSIPIEF**RVSLL**RDCPNKRAIYA 1255

************:*******:* ****************** *************:****

α32 α33

swine 1258 SKAVGEPPLFLGASIFFAIKDAIRAARVQHTDNNTKELFRLDSPATPEKIRNACVDKFTS 1317

bovine 1256 SKAVGEPPLFLGASVFFAIKDAIRAARAQHTNNNTKELFRLDSPATPEKIRNACVDKFTT 1315

cat 1255 SKAVGEPPLFLAASIFFAIKDAICAARAGNPDCKTKKLFQLNSPATPEKIRNACVDQFTR 1314

human 1257 SKAVGEPPLFLAASIFFAIKDAIRAARAQHTGNNVKELFRLDSPATPEKIRNACVDKFTT 1316

mouse 1259 SKAVGEPPLFLASSIFFAIKDAIRAARAQHGDSNAKQLFQLDSPATPEKIRNACVDQFTT 1318

rat 1256 SKAVGEPPLFLASSIFFAIKDAIRAARAQHGD-NAKQLFQLDSPATPEKIRNACVDQFTT 1314

***********.:*:******** ***. : . :.*:**:*:**************:**

β46

swine 1318 LCVTEVPEHCKPW**SLR**V 1334

bovine 1316 LCVTGAPGNCKPW**SL**RV 1332

cat 1315 LCVTGTAESCKPW**SVR**V 1331

human 1317 LCVTGVPENCKPW**SVR**V 1333

mouse 1319 LCATGTPENCKSW**SVR**I 1335

rat 1315 LCVTGVPENCKSW**SVR**I 1331

**.* .. **.**:*:

**Figure S1. Amino acid sequence alignment of porcine XOR with other five mammalian XORs.** Totally conserved residues of XORs in all 6 mammalian species were shown by asterisks (*), partially conserved residues are shown by dots (.or :). The second structures were predicted by PSIPRED V3.0 software (<http://bioinf.cs.ucl.ac.uk/psipred/>). The α-helices are shown in underlined letters, and the β-strands in boldface letters. The universally conserved Glu and Arg in the active sites of Mo-pt center are marked with filled circles (●). The eight key amino acid residues, Gly47, Asn352, Ser360, Arg427, Asp430, Asp431, Ser1227 and Lys1230 (swine numbering), generated by molecular docking, are indicated as numbered underlined letters in bold.
