# Supplementary figures and images for: Mechanism of Porcine Liver Xanthine Oxidoreductase Mediated N-Oxide Reduction of Cyadox as Revealed by Docking and Mutagenesis Studies
Source: PLoS One. 2013 Sep 9;8(9):e73912. doi: 10.1371/journal.pone.0073912 (PMC3767608; doi:10.1371/journal.pone.0073912)

## Slide 1
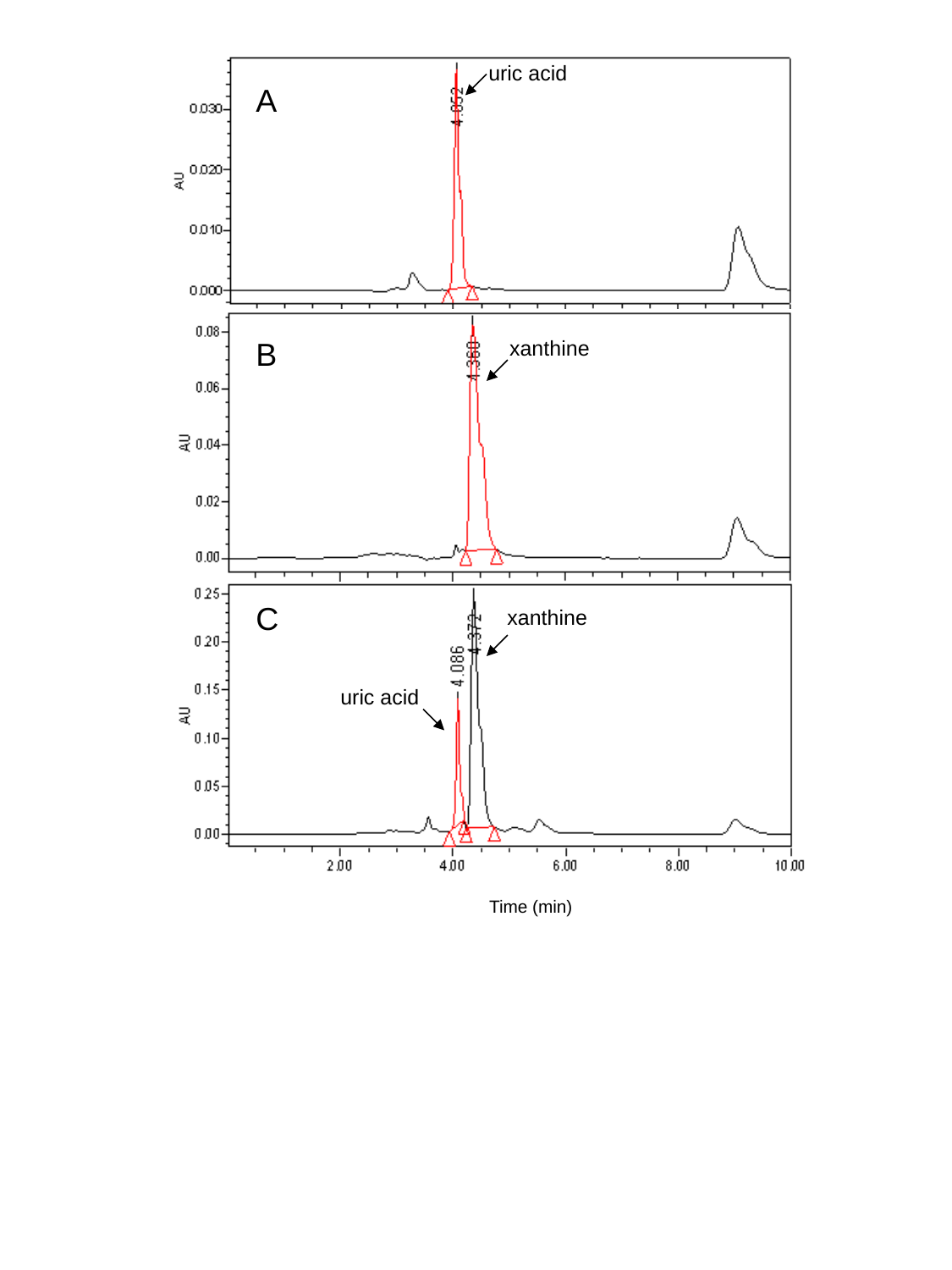

A
B
C
uric acid
xanthine
xanthine
uric acid
Time (min)

Supplement: Figure S2 — HPLC chromatograms of the metabolite of xathine catalyzed by recombinant porcine XOR. (A) Uric standard. (B) Xanthine incubated with Sf9 cell cytosol free of recombinant porcine XOR at 37°C for 20 min. (C) Xanthine incubated with Sf9 cell cytosol containing recombinant porcine XOR at 37°C for 20 min. (PPT) [file pone.0073912.s002.ppt]

## Slide 1
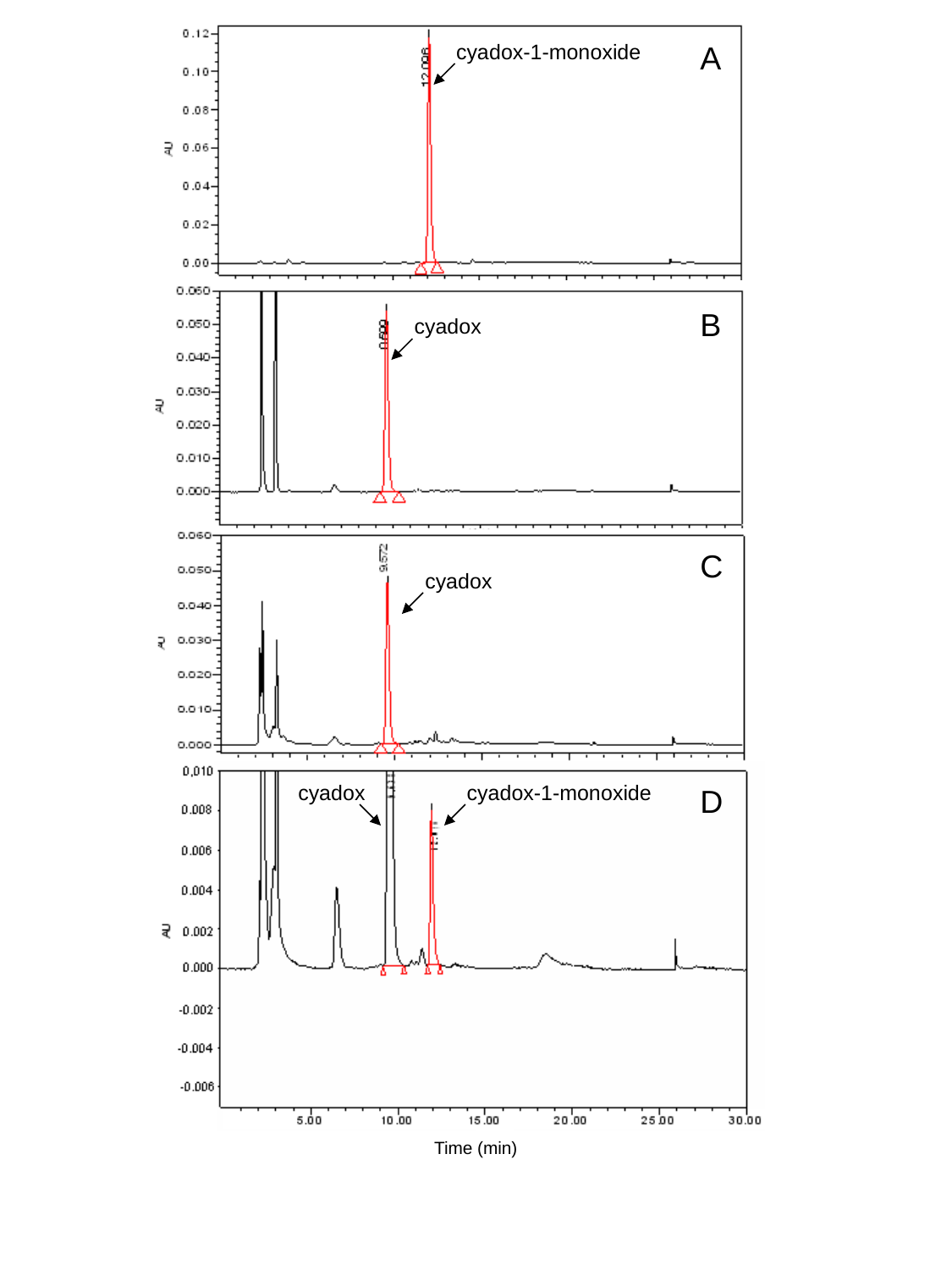

cyadox-1-monoxide
A
B
cyadox
C
cyadox
cyadox
cyadox-1-monoxide
D
Time (min)

Supplement: Figure S3 — HPLC chromatograms of the metabolite of cyadox catalyzed by recombinant porcine XOR. (A) Cyadox-1-monoxide standard. (B) Cyadox incubated with Sf9 cell cytosol free of recombinant porcine XOR with xanthine at 37°C for 60 min. (C) Cyadox incubated with Sf9 cell cytosol containing recombinant procine XOR in the absence of xanthine at 37°C for 60 min. (D) Cyadox incubated with Sf9 cell cytosol containing recombinant porcine XOR in the presence of xanthine at 37°C for 60 min. (PPT) [file pone.0073912.s003.ppt]
